# Role of Obesity, Mesenteric Adipose Tissue, and Adipokines in Inflammatory Bowel Diseases

**DOI:** 10.3390/biom9120780

**Published:** 2019-11-26

**Authors:** Jan Bilski, Agnieszka Mazur-Bialy, Dagmara Wojcik, Marcin Surmiak, Marcin Magierowski, Zbigniew Sliwowski, Robert Pajdo, Slawomir Kwiecien, Aleksandra Danielak, Agata Ptak-Belowska, Thomas Brzozowski

**Affiliations:** 1 Department of Ergonomics and Exercise Physiology, Faculty of Health Sciences, Jagiellonian University Medical College, 20 Grzegorzecka Street, 31-531 Cracow, Poland; mpbilski@cyf-kr.edu.pl (J.B.); agnieszka.mazur@uj.edu.pl (A.M.-B.); 2 Department of Physiology, Faculty of Medicine, Jagiellonian University Medical College, 16 Grzegorzecka Street, 31-531 Cracow, Poland; dagmara1.wojcik@uj.edu.pl (D.W.); msurmiak@cm-uj.krakow.pl (M.S.); m.magierowski@uj.edu.pl (M.M.); AgaZS@poczta.fm (Z.S.); mppajdo@cyf-kr.edu.pl (R.P.); skwiecien@cm-uj.krakow.pl (S.K.); aleksandradanielak26@gmail.com (A.D.); agata.ptak-belowska@uj.edu.pl (A.P.-B.)

**Keywords:** inflammatory bowel disease, Crohn’s disease, ulcerative colitis obesity, adipose tissue, adipokines, inflammation

## Abstract

Inflammatory bowel diseases (IBDs) are a group of disorders which include ulcerative colitis and Crohn’s disease. Obesity is becoming increasingly more common among patients with inflammatory bowel disease and plays a role in the development and course of the disease. This is especially true in the case of Crohn’s disease. The recent results indicate a special role of visceral adipose tissue and particularly mesenteric adipose tissue, also known as “creeping fat”, in pathomechanism, leading to intestinal inflammation. The involvement of altered adipocyte function and the deregulated production of adipokines, such as leptin and adiponectin, has been suggested in pathogenesis of IBD. In this review, we discuss the epidemiology and pathophysiology of obesity in IBD, the influence of a Western diet on the course of Crohn’s disease and colitis in IBD patients and animal’s models, and the potential role of adipokines in these disorders. Since altered body composition, decrease of skeletal muscle mass, and development of pathologically changed mesenteric white adipose tissue are well-known features of IBD and especially of Crohn’s disease, we discuss the possible crosstalk between adipokines and myokines released from skeletal muscle during exercise with moderate or forced intensity. The emerging role of microbiota and the antioxidative and anti-inflammatory enzymes such as intestinal alkaline phosphatase is also discussed, in order to open new avenues for the therapy against intestinal perturbations associated with IBD.

## 1. Introduction

The term inflammatory bowel disease (IBD) refers to a group of chronic, relapsing, and remitting disorders, which are characterized by chronic inflammation of the gastrointestinal tract, and include Crohn’s disease (CD) and ulcerative colitis (UC) [1,2]. Despite the similarities, there are clear differences between these two diseases. The inflammatory process in CD is typically discontinuous, transmural, involving all layers of the gut wall, and although initially described as a disease involving only the terminal ileum, in fact, it could affect the entire digestive tract, from the mouth to the anus. On the other hand, the inflammatory process in UC is continuous but limited to the mucosa and superficial submucosa and may involve only the colon [1,2]. Other features more common in CD than UC are anorexia, altered body composition, and hypertrophy of mesenteric white adipose tissue (mWAT) [1,2]. Mesenteric fat is considered to be a hallmark of CD and was claimed by Dr. Burrill B. Crohn himself to be a consistent symptom of the disease [3]. IBD patients have significantly increased risk of colorectal cancer (CRC), which is probably associated with the consequences of chronic intestinal inflammation [4].

Despite the progress made in recent years in the understanding of IBD pathogenesis, their aetiology is still unclear. One of the theories suggests that a dysregulated mucosal immune response to bacterial components, such as lipopolysaccharide (LPS), could lead to the development of either CD or UC [5,6]. The incidence rates and prevalence of IBD over the past 50 years have increased remarkably in countries that have adapted a “Westernized” lifestyle [7,8], manifested by serious modifications in dietary habits and decreased physical activity. The composition of the gut microbiota is thought to be a critical factor in the development of IBD, and recent studies have shown an association between diet and the composition of the human microbiome [9].

In this review, we provide on update on the epidemiology and pathophysiology of obesity in IBD, the potential effect of total and regional organ obesity with reference to the disease course, the role of adipokines and myokines, and an overview on data from animal experiments.

### 1.1. Epidemiology

The prevalence of overweight and obesity both in developed and developing countries has dramatically increased and is generally considered to be a global pandemic [10]. It is interesting to note that the incidence and prevalence of IBD is also growing in parallel to the obesity pandemic [11,12]. The increasing prevalence of IBD has a major impact on health-care resources and could be connected to changes in peoples’ lifestyles, such as insufficient physical activity and ingestion of a so-called “Western diet”, rich in animal fat and poor in dietary fibre [13,14,15]. Considering the growing prevalence of both IBD and obesity, as well as the interaction between risk factors common for both conditions, epidemiological interaction between them is often postulated. Obesity could also negatively affect the course of disease in other autoimmune and inflammatory diseases [16,17,18,19,20].

Traditionally weight loss and low body mass index (BMI) were commonly considered to be presenting features for IBD [21,22,23], more frequently common and severe in patients with in CD than UC [24,25,26]. As IBD patients were previously considered to be malnourished, their being overweight was relatively rare, either at presentation or during the disease [27]. Recent studies, however, have demonstrated a growing prevalence of obesity in both adult and paediatric IBD patients [28,29,30,31,32,33,34]. In observational studies carried out in Scotland, Steed et al. [31] observed a significant increase in incidence of IBD in obese patients. Among these patients, 18% of the CD population was obese, and a total of 52% was overweight or obese. The authors concluded that this increase confirms the rising prevalence of obesity and overweight in the general population. On the other hand, they observed that only small number of patients were underweight (3% of CD, 0.5% of UC patients). They have also noticed that obesity was significantly more common in CD than UC patients.

A similar phenomenon was observed in paediatric IBD patients (4–16 years old). Long et al. [29] observed that 23.6% of paediatric IBD patients were overweight or obese. They also observed that prior IBD-related surgery was associated with overweight or obesity in these paediatric CD patients.

### 1.2. Obesity as a Risk Factor for the Development of IBD

Despite the increased prevalence of obesity in patients with IBD, the pathomechanism by which obesity affects the course of IBD remains unexplored [35,36,37,38,39,40]. In a large prospective female cohort from the USA (The Nurses’ Health Study), authors found that obesity measured by BMI and body habits are associated with a higher risk of developing CD than UC [35]. A Danish cohort study of 75,000 women (Danish National Birth Cohort) looking for an aetiological link between obesity and certain autoimmune diseases has demonstrated an increased risk of CD (but not for UC) in both underweight and obese women compared with normal-weight women [37]. In the follow-up of this study, authors confirmed the aetiological link between obesity and the risk of CD [41].

Using the Swedish Hospital Discharge Register, Hemminge et al. [42] defined a cohort of patients hospitalized for obesity since year 1964. The patients were followed for hospitalization for selected autoimmune disease through year 2007. The authors observed that the relative incidence of CD was highest when obesity was diagnosed before 30 years of age. In a cohort study of individuals from the Copenhagen School Health Records Register (CSHRR), the authors examined the association between BMI values in childhood (7–13 years) and the later development of IBD. They found that childhood obesity could be a risk factor for CD but underweight might be a risk factor for UC [39]. In contradiction to the above studies, the European Prospective Investigation into Cancer and Nutrition study (EPIC), including more than half a million participants, found that obesity, as defined by BMI, is not associated with the development of UC or CD. The possible reason for these conflicting results is fact that the previously mentioned studies included children or young women as opposed to the EPIC study, which included both men and women and a large percentage of older people. It was suggested that the effect of obesity on risk of CD might be age-dependent, with obesity in young age prompting a higher risk of developing CD in older age [37,41,42].

In their recent meta-analysis, Rahmani et al. [43] demonstrated that obesity is a significant risk factor related to the incidence of CD but not UC. As patients with CD have higher visceral fat volumes (VAT) compared to healthy individuals [44] and visceral adipose compartment is metabolically active and is a possible source of proinflammatory substances [45,46], VAT volume could be more predictive for disease development than overall obesity determined by BMI. In a prospective cohort study, Khalili et al. [35] detected in patients with a high waist–hip ratio (WHR), a trend toward increased risk of CD, but not UC.

### 1.3. Effect of Obesity on the Course of IBD

The impact of obesity on IBD phenotype and outcomes, when assessed by BMI, has not been consistently associated with clinical outcome or disease severity in patients with IBD (Table 1). Blain et al. [27] have shown that adult CD patients with BMI > 30 kg/m^2^ had more frequent perineal complications and more frequent hospitalizations. The retrospective case-control study conducted by Hass et al. [47] reported that obese CD patients (BMI > 25 kg/m^2^) had earlier surgery than nonobese patients. Similarly, paediatric IBD patients with a high BMI had an increased need for surgery [29]. In their retrospective cohort study, Malik et al. [48] found that obese CD patients were approximately 2.5 times more likely to present a poor surgical outcome than those who were nonobese. In a more recent, retrospective study of 209 adult patients with CD, Singla et al. [49] observed that patients with a higher BMI were more likely to present with extraintestinal manifestations. Pavelock et al. [50], in their retrospective observational study on IBD patients (63% CD and 37% UC), found that obesity negatively influences the clinical course of IBD and may increase the burden of disease and treatment. They critically evaluated an increasing trend in needed health care and escalations of various therapies against obesity.

In contrast, Seminerio et al. [51] showed that IBD patients with a high BMI had lower scores on quality-of-life (QoL) metrics, but they did not require additional health-care expenses or more frequent IBD-related surgeries. Flores et al. [52] observed that obese (with a high BMI) IBD patients have less frequent IBD-related surgeries and hospitalization as compared to normal/underweight patients. Pringle et al. [53] observed that obese CD patients have no higher risk of structuring disease, perianal disease, or more frequent surgery compared to nonobese patients, but they presented lower prevalence of penetrating disease complications. Similarly, in UC patients, the higher BMI has not been associated with disease severity. In a cohort of 202 patients with UC, Stabroth-Akil et al. [54] observed that a chronic active disease was less prevalent in obese patients than in those with normal weight.

Singh et al. [55] presented the data from a pooled analysis of placebo controlled clinical trials with infliximab and found that obesity assessed by BMI does not significantly influences short- and intermediate-term clinical outcomes in patients with IBD. Recently, Hu et al. [56] performed a meta-analysis to assess the association between obesity defined by BMI >30 kg/m^2^ and clinical outcomes in IBD patients and found that obesity was associated with a less-severe disease course of IBD. A number of authors have pointed out that reliance on BMI as a sole marker of obesity seems to be the serious limitation of studies on relationship between IBD and obesity. They indicated a poor linear relationship between BMI and total body fat and also suggested that body fat distribution would be more clinically significant than overall obesity [43,50,56,57].

Studies in patients with CD disease using visceral adiposity as a measure of obesity have more consistently shown the increased risk of CD-related complications than those using BMI as a marker of overall obesity [58,59,60,61,62,63,64]. Erhayiem et al. [58], in a study on 97 patients with CD, found that using computed tomography (CT) scanning that mesenteric fat index (MFI), defined as the ratio of areas of visceral-to-subcutaneous fat was a good marker of aggressive CD. These observations were confirmed in study by Li et al. [59] also using CT scanning method; they found that visceral fat area and MFI values were associated with postoperative recurrence of Crohn’s disease. Bryant et al. [60], in a prospective study on 97 patients with CD, used dual energy X-ray absorptiometry (DXA) as a method to assess VAT. They also reported that VAT/subcutaneous adipose tissue [SAT] ratio, rather than BMI, was associated with structuring CD behaviour, an increase in disease activity, and reduced QoL.

The visceral/subcutaneous adipose tissue ratio measured by CT scanning constitutes a better and more reliable predictor of postoperative outcomes in CD patients undergoing ileocolectomy than BMI [61]. Similarly, Holt et al. [62] reported that visceral adiposity measured by CT is an independent risk factor for endoscopic recurrence of Crohn’s disease after surgery. In another study on CD patients, CT scanning was found to be superior to BMI, and VAT volume was considered to be a useful variable and an indicator of increased risk of surgery and penetrating disease. They concluded that visceral, rather than total, adiposity may negatively influence the long-term risk of progression of CD [63].

### 1.4. Skeletal Mass Depletion in IBD

In many patients with IBD and particularly with CD, the body composition, reflected by as proportions of bone, fat, and lean body mass may be abnormal. Sarcopenia, defined as depletion of muscle mass and impaired muscle function [65,66,67,68,69,70,71,72,73,74,75,76,77,78,79,80,81,82], is an important feature in this disease [70,71,72,81]. Depletion of lean body mass and loss of muscle strength associated with lower QoL and higher mortality and morbidity commonly occurs as part of the aging process [65,77]. However, these disorders are also characteristic for malnutrition and chronic intestinal inflammation such as IBD [77].

Recently, a number of reports about the increasing rates of sarcopenia in patients with IBD, especially in patients with CD were observed [69,78]. Such sarcopenia in IBD patients is associated with an increased risk of surgery, poor surgical outcomes, lower QoL, osteopenia, and easy fatigue [66,67,70,71,72,73,74,77,80]. The unchanged or elevated BMI was observed in IBD patients who suffered with loss of muscle mass, followed by muscle impaired function [68,70,71,72]. Recent papers suggested the necessity for the body composition assessment and muscle strength (e.g., by isometric handgrip strength) examination of all IBD patients, and not only those visibly malnourished [66,70,77].

In their prospective study, Bryant et al. [70] reported on 154 patients, using DXA, that raised rates of obesity in patients with IBD, and these effects coincided with depletion of skeletal muscle mass over time. Furthermore, faecal calprotectin as a measure of disease activity and intestinal inflammation was negatively correlated with skeletal mass index. Isometric handgrip strength in those patients was positively associated with skeletal mass index and negatively with fat mass index [70].

It was proposed that the important causative factor in skeletal muscle wastage in patients with CD could be the local and systemic inflammation caused, in part at least, by proinflammatory cytokines released from hypertrophied visceral adipose tissue [75,83,84,85,86].

## 2. Obesity in the Pathogenesis of IBD

### 2.1. Obesity and Inflammation

Obesity is associated with a low-grade chronic inflammatory state, characterized by the activation of proinflammatory signalling pathways, increased synthesis of acute-phase reactants, such as C-reactive protein (CRP), and increased proinflammatory cytokines production [87]. Activation of the proinflammatory transcription factor NF-κB in adipocytes is a common finding in obese subjects [88]. At present, adipose tissue is considered not only as an inert storage organ, but also an endocrine organ that synthesizes a number of biologically active substances called adipokines, such as adiponectin (APN), IL-1, IL-6, IL-8, IFNγ, TNF-α, leptin, apelin, chemerin, and resistin [88]. Adipokines can regulate metabolic homeostasis and affect immune functions [89].

**Figure 1:**
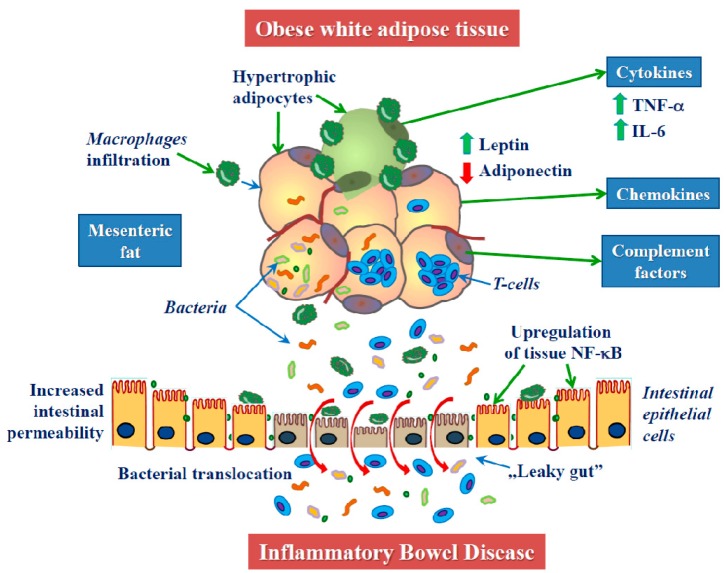
Mechanisms linking obesity with IBD. Mesenteric fat deposition in obese individuals leads to hypertrophic adipocytes releasing various proinflammatory cytokines, chemokines complement factors, and the disturbance of immune homeostasis in the intestine. This can directly and indirectly participate in low-grade inflammation, imbalance between leptin–adiponectin ratio, the disruption of intestinal mucosa and the induction of intestinal permeability, which in turn enhance fat-derived inflammatory adipokines, bacterial translocation, and the stimulated T-cell infiltration, considered as “leaky gut”—thus predisposing to IBD. Tumor necrosis factor α (TNF-α), nuclear factor kappa-light-chain-enhancer of activated B cells (NF-κB), interleukin 6 (Il-6)

Adipose tissue is far from being uniform, and there are two major types: white adipose tissue (WAT) and brown adipose tissue (BAT) [91]. In recent years, the third type was postulated—beige (or bright) adipose tissue [92]. WAT is divided into two distinct depots: visceral (VAT) and subcutaneous adipose tissue (SAT), which display different metabolic and immunological profiles [46,93,94]. Visceral obesity, which has been particularly related to a proinflammatory state, has been implicated in several gastrointestinal diseases, including fatty liver, cancers, acute pancreatitis, and CD [95]. The adipose tissue depots can be pathologically changed due to inflammatory diseases such as CD. The infiltration of adipose tissue by macrophages is characteristic for obesity and leads to increased production of additional inflammatory mediators [93,94,96,97,98] (Figure 1).

The intestinal barrier defects and increased jejunal permeability were reported by Genser et al. [99] in severely obese subjects. Moreover, these obese patients have decreased tight junction proteins occludin and tricellulin, but LPS, LPS-binding protein (LPSB), and zonulin were increased as compared to the control. In the same study, the ex vivo experiments on epithelial cells from obese patients demonstrated that their exposure to dietary lipids to a greater extent compromised the intestinal barrier [99].

### 2.2. Mesenteric White Adipose Tissue in CD

**Figure 2:**
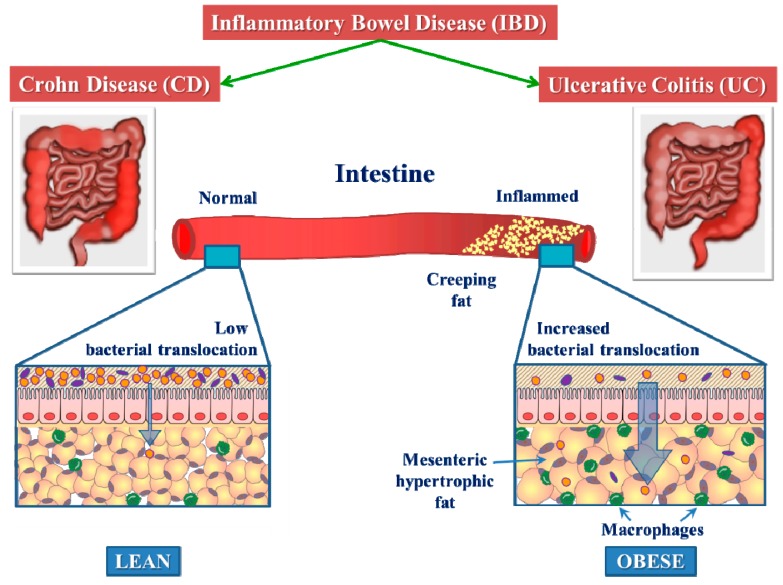
The accumulation of visceral “creeping fat” in IBD of obese individuals causes local intestinal inflammation. The responsible mechanisms are the excessive immune response, as reflected by a greater number of macrophages, and the release of proinflammatory cytokines, leading to increased bacterial translocation (thick arrowhead), as compared with lean individuals.

CD is characterized by the marked alteration in mesenteric adipose tissue properties [100]. In patients with CD, the ratio of intraabdominal fat to total abdominal fat is far greater than in controls, when assessed by magnetic resonance imaging (MRI) [64]. Creeping fat in CD patients refers to pathologically altered mesenteric fat tissue located around the inflamed parts of the intestine [101]. Furthermore, mWAT actively contributes to the disease severity and may influence the onset of complications [98,100,101,102,103,104]. In patients with CD, the localization of mucosal ulcerations is most pronounced along the mesenteric attachments, which suggests a causal link between mesenteric adipose tissue and mucosal changes. In these patients, a selective enlargement of fat depots around the diseased lymph nodes and intestine can be observed, with more than 50% of the intestinal surface covered by fat tissue [105]. Creeping fat can be distinguished from normal mesenteric fat-tissue by its distinctively larger size, and by its greater immune cell infiltration [46,98,106] (Figure 2).

Pathologically altered, the mWAT plays an important function as a source of inflammatory factors, such as cytokines and chemokines [45,98,100,102,103,107,108,109,110,111,112,113]. Creeping fat is thought to be immunologically more active than other VAT, and the extent of creeping fat correlates closely with the extent of the histological inflammation and degree of lymphocyte or macrophage infiltration [114]. The creeping fat is a major source of the increased TNF-α, IL-6 and other circulating proinflammatory cytokines seen in IBD patients. These fat-releasing cytokines may contribute to the debilitating systemic symptoms observed in these patients [100,103]. In pathologically altered mWAT adjacent to the intestinal wall of patients with CD, the higher expression of the hypoxia-inducible factor 1α (HIF-1α) and a decreased number of vessels per adipocyte is observed, which may suggest the role of HIF-Iα in this process [115]. Sideri et al. observe that the preadipocytes isolated from mWAT in IBD patients released IL-17 in response to SP [111]. The mWAT is an important source of CRP in CD patients, and its production by mesenteric adipocytes may be triggered by local inflammation and bacterial translocation to mWAT [102,116].

It is suggested that, in CD disease, the transmural inflammation facilitates increased bacterial translocation into the creeping fat (Figure 2). Translocalizing antigens can directly activate (pre)adipocytes via innate receptors [110,117,118]. Adipocyte-derived mediators modulate the phenotype and function of innate and adaptive immune cells. Adipocytes and preadipocytes express receptors of the TLR family and, for instance, the rise in TLR-4 expression in adipocytes and preadipocytes by LPS activating NF-κB pathways leads to the increased production of classic cytokines and chemokines, including IL-6, MCP-1, and TNF-α [110]. Preadipocytes can additionally differentiate into macrophages [119,120].

Another interesting aspect is the presence of neuronal hyperplasia in patients with CD. The nerve fibres in these patients seem to contain an increased amount of vasoactive intestinal polypeptide (VIP) and substance P (SP) [121]. The potential involvement of neuropeptides, and particularly SP, in IBD pathophysiology has been also proposed [100,111,122,123,124,125]. Human mesenteric preadipocytes contain functional SP receptors which could be linked to proinflammatory pathways, and mWAT may participate in intestinal inflammatory responses via SP–NK-1R-related pathways.

Anorexia is another feature present in CD which could be explained by cytokine overproduction by mWAT [126]. It is generally accepted that reduced food intake may occur in CD and can lead to abdominal pain, fear of diarrhoea and incontinence, surgery, nausea, and depression. Satiety control in these patients could be modulated by inflammatory cytokines, which generally may suppress appetite [126].

Some researchers hypothesize that the mesenteric adipose tissue might serve as a barrier to bacteria, which may have breached the intestinal mucosa and/or play an anti-inflammatory role [46]. The observation that there is an increase of M2 macrophages in the mesentery of CD patients [106] supports the hypothesis of the protective role of the mesentery in this disease. However, recent findings [111,114,127,128] seem to indicate that mWAT in patients with CD exerts rather a proinflammatory actions.

### 2.3. Adipokines

**Figure 3:**
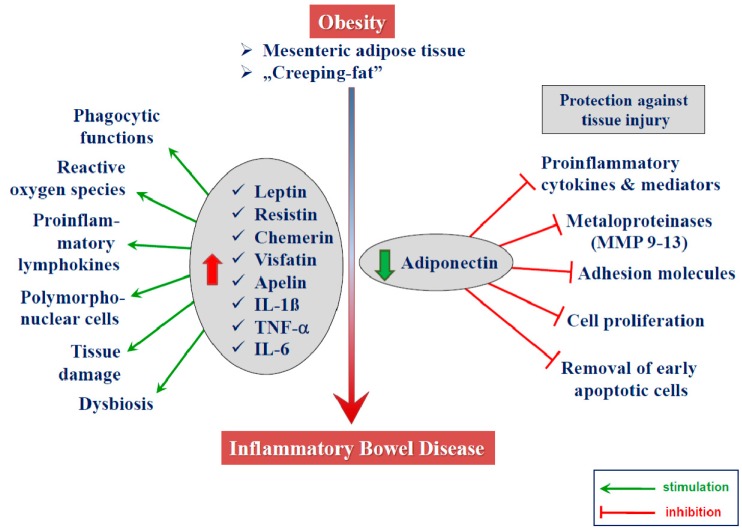
Involvement of adipokines released from creeping fat in IBD. The mesenteric adipose tissue of patients with IBD presents an inflammatory profile with an increased expression of cytokines (e.g., TNF-α, IL-1β, and IL-6) and adipokines (e.g., leptin, resistin, chemerin, and visfatin) involved in intestinal inflammation. In contrast, beneficial adipokine adiponectin, which has been shown to inhibit the expression of adhesion molecules, metalloproteinases, and proinflammatory mediators, is downregulated in IBD. This downregulation contributes to the pathogenesis of these intestinal disorders.

When linking obesity and inflammatory processes in IBD, adipokines are of particular interest. In several pathological states, the strong correlation between adipokine levels and inflammation severity is demonstrated [57,90,129,130,131] (Figure 3). However, the results of the discussed studies are contradictory, as there is still no consensus on the exact role they play in the pathogenesis and course of IBD.

#### 2.3.1. Leptin

Leptin is mainly secreted by white adipose tissue in response to the amount of body fat in a pulsatile fashion and with a diurnal variation. The most important function of leptin is the regulation of the energy homeostasis and metabolism. Leptin exerts a strong proinflammatory effect on the immune system and can be released in response to inflammatory stimuli, such as interleukin-1 (IL-1), IL-6, LPS, or bacterial infection [99]. Due to leptin acting as a proinflammatory adipokine, especially in obese subjects, this peptide is implicated in the pathogenesis of IBD, and therefore leptin antagonists are postulated as a potential therapeutic option for IBD [132,133]. However, the results of the clinical studies examining the serum leptin levels in IBD are ambiguous. In a recent systematic review on adipokines in IBD, no linear association between leptin levels and IBD severity was demonstrated [129].

Biesiada et al. found that leptin levels in patients with an exacerbation of UC are higher than in those in remission, and these values of leptin correlate positively with serum levels of proinflammatory cytokines IL-1β and TNF-α—but not with the severity of the inflammatory intestinal lesions [134]. A similar observation was made by Tuzun et al., who found elevated levels of leptin in patients in the acute stage of UC [135]. In another study, Kahraman and colleagues [136] showed that leptin levels were much lower in patients with UC and CD than in healthy controls. In contrast, Karmiris et al. [137] observed that serum levels of leptin were reduced in patients with IBD. There are no differences between the patients with CD or UC, or between patients with active or inactive disease. A similar observation was made in pediatric IBD patients who presented reduced leptin levels [138], and there was also no difference between patients in remission and active disease. On the other hand, UC patients in remission have significantly higher leptin than those patients with active disease. Other studies, however, do not find any changes in the level of leptin levels in IBD patients comparing to the control [139,140,141]. The inconclusive observations and differences between these studies could be explained by the small numbers of patients used and the different controls and treatments which are employed.

When the expression of leptin in mesenteric fat in IBD patients is studied, the results are more conclusive. Barbier et al. [142] and Paul et al. [143] reported the overexpression of leptin mRNA in mesenteric adipose tissue in IBD (both CD an UC) patients in comparison to healthy intestinal specimens [143].

#### 2.3.2. Adiponectin

Adiponectin (APN) is a more abundant adipocyte-specific adipokine which exhibits an anti-inflammatory action and plays a key role in the regulation of insulin sensitivity. The APN concentrations in obese subjects are lower than in normal weight controls (Figure 3). Similar to leptin, the data concerning APN serum levels in patients with IBD is controversial. For instance, Kahraman et al. [136] find that, unlike in other studies, serum APN concentrations are decreased in both UC and CD patients. Similarly, Valentini et al. [140] demonstrated that APN serum levels are decreased in active and inactive disease in both CD and UC individuals. In contrast, Karmiris et al. [137] reported that serum levels of adiponectin are increased, whereas serum levels of leptin are decreased, in patients with IBD. Weigert et al. [144] observed that patients with CD had lower APN serum levels in comparison to UC, that APN is lower in female CD patients in comparison to female healthy controls, and that APN reaches higher serum levels in UC patients in comparison to healthy controls. However, in other studies, there were no significant changes in the APN level in IBD patients in comparison to the controls [138,145].

The study by Yamamoto et al. [146] revealed an upregulation of adiponectin expression in creeping fat of CD patients in comparison to normal mesenteric adipose tissue of CD patients, as well as mesenteric fat from UC patients or controls. A similar observation in creeping fat of patients with CD was reported by Paul et al. [143]. In contrast, Rodrigues et al. [139] observed that APN expression in mesenteric fat is lower in patients with active ileocecal CD in comparison to the controls.

#### 2.3.3. Other Adipokines

Han et al. [147] observed increased colonic apelin production in both UC and CD patients. Ge et al. [148] demonstrated that apelin is highly expressed in the mesenteric fat of patients with CD and suggested that apelin, which is essential for the development and the stabilization of lymphatic vessels, could play a supportive role with regard to intestinal lymphatic drainage in CD.

Chemerin is an adipokine acting as a chemo-attractant for cells of the innate immune system and has been linked with several inflammatory conditions. Higher levels of serum chemerin in IBD patients are observed in some [144,149], but not all [145], studies.

Resistin, originally described as an adipocyte-specific hormone, is expressed and secreted from macrophages in humans, and it exerts a strong proinflammatory action. Resistin is implicated in the pathogenesis of obesity and insulin resistance [150]. Resistin serum levels are commonly elevated in inflammatory conditions, such as IBD [137,151,152], and are significantly decreased after infliximab therapy in IBD patients [152].

Another adipokine, visfatin, can play a significant role in the intracellular and extracellular metabolic effects associated with obesity [150]. The levels of visfatin are strongly correlated with the amount of visceral fat and mesenteric adipose tissue [150]. Serum visfatin levels are increased in IBD patients [145,149,153,154], and a higher expression of visfatin is found in colonic biopsies of IBD patients [154,155]. The correlation between visfatin levels in the colonic biopsies with disease activity is also observed in paediatric IBD patients [154].

Vaspin belongs to family of newly discovered adipokines besides others such as retinol-binding protein 4 (RBP4), dipeptidyl peptidase 4 (DPP-4), bone morphogenetic protein (BMP)-4, BMP-7, and progranulin, recently implicated in various aspects of obesity [156]. For instance vaspin is a newly discovered adipokine with insulin-sensitizing and anti-inflammatory effects [157,158]. Terzoudis et al. [149] find no difference in the serum concentrations of vaspin between IBD patients and healthy controls. In contrary to this observation, Morisaki et al. [157] report that serum vaspin levels are higher in patients with IBD than in controls. The authors additionally observe that vaspin is expressed in the adipocytes of the mesenteric WAT in IBD patients.

Recently, omentin-1, also known as intelectin-1, was not only identified in the visceral (omental) fat, but also in the small intestine, colon, ovary, and plasma [158]. In addition to its anti-inflammatory action, omentin-1 plays an important role in the homeostasis of the body metabolism and in insulin sensitivity [158]. Yin et al. [159] observed significantly decreased serum omentin1 levels in patients with IBD, in comparison to healthy controls. Similarly, Lu et al. [160] reported that serum omentin-1 levels and colonic omentin-1 expressions are reduced in active CD patients, in addition to their correlation with disease activity.

Meteorin-like (Metrnl) is a new adipo-myokine, highly expressed in WAT. This adipo-myokine is induced in skeletal muscle upon cold exposure, and this peptide has been shown to exert an anti-inflammatory activity due to an increase in beige fat thermogenesis [161,162]. Metrnl expression is higher in mWAT of CD patients in comparison to the controls [163].

In conclusion, present findings on the role of various adipokines in IBD are inconsistent, and human studies with a larger number of patients and more uniform methodology are needed (Table 2).

### 2.4. Dietary Links with IBD

Epidemiological studies suggest that both the development of obesity and IBD could generate a proinflammatory state through the expression and release of inflammatory cytokines and chemokines in response to the so-called Western diet [164,165]. A Japanese study investigated a possible link between the transition from a traditional diet to a high-fat Western diet, and increased incidence of CD [166]. In this study [165], the CD incidence is strongly correlated with an increased dietary intake of total fats, animal fat, n-6 polyunsaturated fatty acids (PUFA), and animal and milk protein. The systematic review by Hou et al. also demonstrated an association between an increased CD or UC risk and a high intake of PUFAs, omega-6 fatty acids, saturated fats, and meat [167].

Accumulating evidence indicates that the composition of the gut microbiota plays a critical role in the development of obesity, obesity-associated inflammation, and IBD, representing another common link in the pathogenesis of these conditions [168,169,170,171,172]. Patients with IBD have demonstrated intestinal dysbiosis, which is defined as a decrease in gut microbial diversity [173]. Such a fall in bacterial diversity and dysbiosis is characterized by the reduction of *Firmicutes* and the rise of *Bacteroidetes* and *Proteobacteria* [2,168].

Dysbiosis caused by the Western diet rich in sugar and fat may lead to a dysfunction of the intestinal mucosal barrier, increased permeability, and bacterial translocation, which are common features of obesity and IBD pathogenesis [110,174,175]. Translocalizing antigens can directly activate adipocytes and preadipocytes, with subsequent increased release of proinflammatory cytokines; possibly leading to a positive feedback loop that enhances inflammation [110,117,118]. A marked correlation between a high-fat diet and elevated markers of bacterial translocation, such as LPS, LBP, and TLR-4, throughout “leaky gut” has been demonstrated [176,177,178].

## 3. Experimental Studies on Role of Adipose Tissue in IBD

Since the data concerning links with IBD and obesity in humans are inconclusive, various models of experimental colitis have been used to study this relationship. Animal studies could provide a better insight into the potential mechanisms through which adipose tissue could exert its effects on the course of the disease. Numerous studies confirm that a high-fat diet (HFD) or high-fat and high-sugar diets (HF/HSD) can exacerbate experimentally induced colitis. In murine colitis models, the diet modifications are attributed to the alterations in the plasma levels of proinflammatory biomarkers and the expression of proinflammatory factors in VAT [102,108,128,179,180,181,182,183,184,185,186,187]. It was demonstrated that the application of an HFD or HF/HSD diets and/or the development of obesity in mice increase(s) the intestinal permeability and bacterial translocation from the intestinal lumen to mesenteric fat, as well as profound changes in the microbiota [102,108,127,181,184,185,186,187,188,189,190,191,192,193,194,195]. HFD or HF/HSD is also associated with significantly elevated LPS levels, the reduced expression of epithelial tight junction proteins, an increased macrophage infiltration, and the increased expression of proinflammatory biomarkers in the adipose tissue [102,127,181,184,185,186,187,188,189,190,191,192,193,196,197,198]. Both, the Paneth cell area and the release of antimicrobial factors by Paneth cells are reduced in HFD-fed mice [197]. The increase in endoplasmic reticulum (ER) and oxidative stress, impaired mucosal barrier integrity, and rise in biomarkers increase serum LPS levels in HFD-fed mice [195]. Similarly, in the same study, non-esterified long-chain saturated fatty acids increase oxidative and ER stress in cultured intestinal cells. Collectively, these data demonstrate that a diet which mimics Western eating habits can promote inflammation and ER stress and increases intestinal permeability. Moreover, the HFD resulting in the mesenteric fat in these animals induces alterations in gut microbiota reminiscent of the pathological phenomena in CD patients. However, it is still under debate whether the observed effects are associated with fat accumulation and pathologically altered adipose tissue leading to obesity or caused by the diet alone affecting microbiota [199,200,201,202]. Gruber et al. [202] reported the effect of HFD on the development of chronic ileal inflammation in a TNF^ΔARE/WT^ mice genetic mouse model of Crohn’s disease-like ileitis, and they found that HFD, independent of obesity, exacerbated small intestinal inflammation. In an interesting paper by Bibi et al. [203], it was demonstrated that maternal HFD predisposes offspring to a higher susceptibility to developing experimental DSS-induced colitis.

Experimental animal studies have also allowed for better insight into the role of adipokines in the course of colitis. Siegmund et al. [204] et al. induced experimental colitis, using dextran sulphate sodium (DSS) or trinitrobenzene sulfonic acid (TNBS) in leptin-deficient ob/ob mice. Leptin deficient mice have significantly reduced colitis severity and release of proinflammatory cytokines from the colon, in comparison to wild-type (WT) mice. The administration of leptin to ob/ob mice leads to a similar disease severity and proinflammatory cytokine production, as observed in WT mice. However, the administration of leptin to control WT mice does not significantly influence the severity of the disease [204]. IL-10-deficient (IL-10^−/−^) mice show spontaneous development of chronic intestinal inflammation [205]. In a study by the same group [206], the leptin-deficient IL-10^−/−^ mice model was introduced to evaluate the role of leptin in a model of spontaneously developing inflammation. The study observed that, in both IL-10^−/−^ ob/ob and in IL-10^−/−^ mice, a similar degree of intestinal inflammation develops [206]. It is concluded that leptin does not play a significant role in the spontaneous colitis of IL-10^−/−^ mice. On the other hand, Singh et al. [133] observed that pegylated leptin antagonist ameliorated chronic colitis in IL-10^−/−^ mice.

In another study [185], authors observed that the impaired healing of TNBS-induced rats fed HFD is accompanied by an increase in plasma levels of leptin and a reduction in adiponectin levels. Furthermore, leptin expression is elevated and adiponectin decreased in adipose tissue in rats with colitis fed with a normal diet, and this effect is markedly enhanced in rats fed with an HFD diet. This observation is further supported in studies in mice because the increased leptin and decreased adiponectin plasma levels and elevated expression of leptin and decreased adiponectin expression are recorded in adipose tissue, along with a disease exacerbation in mice fed with an HFD diet [4,182] (Table 3) Interestingly, adiponectin-knockout (APN-KO) mice present as more severe in comparison to WT mice [207,208,209]. Adenovirus-mediated supplementation of APN significantly attenuates the severity of colitis in both APN-KO and WT mice [207,210]. The APN-KO mice with DSS induced colitis have a marked increase in AdipoR1 protein, whereas AdipoR2 is reduced in comparison to controls. In in vitro studies, APN reduces apoptotic, anti-proliferative and stress signals in HCT116 colonic epithelial cells. The abrogation of AdipoR1 promotes apoptosis in in vitro models [210]. The hypothesis on the protective role of adiponectin in colitis, acting through AdipoR1, is supported by the evidence from Sideri et al. [127], who showed that intracolonic AdipoR1 knock down worsened TNBS-induced colitis in mice. In contrast, some studies [211,212] report that APN absence protects against DSS induced colitis. In another study, APN deficiency did not significantly modulate the inflammation in the IL-10 KO model of spontaneous chronic colitis [213].

Han et al. [147] reported increased colonic apelin production in rats and mice with DSS-induced colitis. Ge et al. [148] demonstrated that apelin significantly ameliorates chronic colitis in IL-10^−/−^ mice, as demonstrated by the decreased disease activity index and inflammatory scores. In IL-10^−/−^ mice with spontaneous colitis, the administration of a new adipokine, metrnl, decreased pathological alterations in mWAT, increased adipocyte size and ameliorated inflammation [164].

Recently, in an interesting study, Hoffman et al. [214] demonstrated that mesenteric adipose-derived stromal cells from CD patients could exert beneficial protective effects on the disease activity and severity of mice with experimental colitis. Because of the potential pathogenic role of adipose tissue and adipokines in development of IBD, some experimental studies attempt to reduce colitis severity by reducing the total or particular organ obesity.

Li et al. [215] investigated the effect of the role of telmisartan on pathologically altered mWAT in IL-10^−/−^ mice with spontaneous colitis, with a major aim to analyze the inflammatory response and adipokine production. Telmisartan acts as the antagonist of receptor angiotensin II type 1 and also as a partial agonist of peroxisome proliferator-activated receptor γ (PPAR-γ) [216]. This latter aim is selected because the PPAR-γ activation reduces the severity of experimental colitis [217,218,219]. Telmisartan is shown to reduce the visceral adiposity due to attenuation of leptin and increasing APN expression in adipose tissue in addition to increasing APN serum levels [220,221]. In their study [215], the treatment with telmisartan has ameliorated spontaneous colitis and reduces the pathological changes in mWAT. This effect was associated with lower production of proinflammatory cytokines. Additionally, mice receiving telmisartan have reduced leptin and increased adiponectin mRNA expression in mWAT [215]. Interestingly, both bariatric surgery and, particularly, the duodenojejunal bypass have ameliorated the severity of colitis in chemically induced IBD [222].

Skeletal muscle wastage has been widely observed in patients with CD [69,78] and the role of skeletal muscle—adipose tissue crosstalk in this disease—has been postulated [84,86]. The hypothesis is moved forward that exercise may exert the protective effect, particularly in experimental colitis exacerbated by HFD. This beneficial effect of exercise is to some extent mediated via muscle-derived peptides, so-called “myokines” with endocrine effects, exerting a direct anti-inflammatory action, and/or specific effects on visceral fat [84,192,223,224,225]. Liu et al. [187] proposed an alternative explanation for the protective action of voluntary exercise in HFD-fed mice. The sedentary mice that were fed an HFD diet showed an increased expression of inflammatory mediators and activation of NF-κB in the colon. These changes are associated with the decreased expression and activity of PPAR-γ, and the reversal of these changes are observed by voluntary physical exercise. However, the administration of a selective PPAR-γ antagonist blocks all these beneficial effects [187], indicating that the PPAR-γ system exhibits a protective action in IBD and could be considered to be an important regulator of intestinal integrity in inflamed bowel diseases.

## Figures and Tables

**Table 1:** Effect of obesity on IBD course.

Reference	Year	Study Design	Sample	Marker of Obesity/Overweight	Conclusion
Blain et al. [27]	2002	Retrospective	2065 CD patients	BMI ≥ 25.0 kg/m^2^ at disease onset and BMI > 30.0 kg/m^2^ anytime during the course of the disease	Obesity was associated with more frequent anoperineal. complications and more marked year-by-year disease activity, but does not alter significantly the long-term course of the disease.
Hass et al. [47]	2006	Cross-sectional	148 CD patients	BMI ≥ 25.0 kg/m^2^	Patients with a BMI > 25 kg/m^2^ had a shorter time to first surgery than those with a BMI of less than 18.5 kg/m^2^.
Long et al. [29]	2011	Cross-sectional	1598 children with IBD	BMI	Obese IBD patients have an increased need for surgery.
Erhayiem et al. [58]	2011	Retrospective	50 CD patients	CT scans, MFI defined as the ratio of areas of VAT to SAT	MFI was significantly higher in patients with complicated (strictures and fistulas) disease.
Malik et al. [48]	2013	Retrospective	90 CD patients	BMI ≥ 30.0 kg/m^2^	Obese CD patients had a poor surgical outcome when compared to not obese CD patients.
Connelly et al. [61]	2014	Retrospective	143 CD patients after elective ileocolectomy	CT scans BMI	The VAT/SAT ratio was a predictor of increased risk for postoperative complications in patients after elective ileocolectomy.
Seminerio et al. [51]	2015	Retrospective	1494 IBD patients	BMI ≥ 30 kg/m^2^	Obesity was not associated with increased health-care utilization and IBD-related surgeries.
Flores et al. [52]	2015	Retrospective	581 IBD patients (297 CD and 284 UC).	BMI ≥ 30 kg/m^2^	Obese IBD patients were less likely to have need for anti-TNF therapy, surgery or hospitalization than normal or underweight patients.
Pringle et al. [53]	2015	Cross-sectional	846 patients with CD	BMI ≥ 30 kg/m^2^	There were no associations between obesity and risk of perianal disease, structuring disease, or surgery. Compared with normal-weight individuals, obesity was associated with lower risk of penetrating disease.
Stabroth-Akil et al. [54]	2015	Retrospective	202 UC patients		High BMI had a favourable effect on the prognosis; low BMI pointed to a more severe course of the disease.
Li et al. [59]	2015	Retrospective	117 CD patients after ileocolic resection	CT scans	High visceral fat area value was associated with higher postoperative recurrence, defined as the reappearance of the clinical manifestations of Crohn’s disease.
Van Der Sloot et al. [63]	2016	Prospective	482 patients	CT scans	VAT volume was associated with an increased risk of surgery and penetrating disease but not structuring or perianal disease among CD patients.
Singla et al. [49]	2017	Retrospective	209 CD patients	BMI	Patients with higher BMI were more likely to have extraintestinal manifestations.
Holt et al. [62]	2017	Prospective	44 post-operative Crohn’s disease patients	CT or MRI scans. Waist circumference BMI	Excessive visceral adiposity was an independent risk factor for endoscopic recurrence of Crohn’s disease after surgery. Lower skeletal muscle area correlated with increased fecal inflammatory markers.
Singh et al. [90]	2018	Post hoc analysis	575 IBD placebo-treated patients (pooled analysis of placebo arms, using data from clinical trials of infliximab in IBD)	BMI ≥ 30 kg/m^2^	Obesity does not significantly impact short- and intermediate-term clinical outcomes in patients with IBD.
Pavelock et al. [50]	2019	Retrospective	55 IBD patients (27 CD, 18 UC)	overweight BMI ≥ 25.0 kg/m^2^ obese BMI > 30.0 kg/m^2^	An increasing trend in mean number of clinic visits, hospitalizations/flares, and mean escalations in therapy with an increase in BMI.
Bryant et al. [60]	2019	Prospective	97 CD patients	DXA, BMI, WHR	VAT was associated with structuring CD behavior and prospective disease activity and QoL in a disease-distribution-dependent manner.

Crohn’s disease (CD), ulcerative colitis (UC) Body mass index (BMI), computed tomography (CT), mesenteric fat index (MFI), subcutaneous adipose tissue, visceral adipose tissue (VAT), subcutaneous adipose tissue (SAT), waist/hip ratio (WHR), dual-energy X-ray absorptiometry (DXA).

**Table 2:** The studies examining the potential role of adipokines in IBD.

Reference	Year	Sample	Conclusion
Barbier et al. [142]	2003	19 IBD patients	Leptin mRNA levels are significantly higher in mWAT of CD and UC patients than in controls.
Tuzun et al. [135]	2004	29 patients with active UC	Serum leptin levels are significantly higher in patients with acute UC in comparison to controls.
Nishi et al. [141]	2005	28 CD patients	There are no differences in the plasma leptin levels between CD patients and healthy controls.
Yamamoto et al. [146]	2005	30 IBD patients	Tissue concentrations and release of APN are significantly increased in pathologically altered mWAT in CD patients in comparison to paired normal mWAT from the same subjects. APN mRNA levels are significantly higher in pathologically altered mWAT of CD patients than with normal mWAT of the same CD patients.
Paul et al. [143]	2006	10 CD patients	The secretion of APN and leptin is significantly upregulated in mWAT specimen.
Karmiris et al. [137]	2006	100 IBD patients	Serum levels of adiponectin, resistin, and active ghrelin are higher and serum levels of leptin are lower in patients with IBD than in healthy controls.
Han et al. [147]	2007	IBD patients	In IBD patients, apelin immunostaining demonstrates elevated intestinal apelin content.
Moschen et al. [154]	2007	74 IBD patients	In IBD patients, the plasma visfatin levels are significantly higher and visfatin mRNA expression is significantly elevated in colonic tissue in comparison to healthy controls.
Valentini et al. [140]	2009	128 IBD patients	There are no differences in serum leptin levels between IBD patients and healthy controls. Serum resistin and visfatin concentrations are elevated in patients with active disease, but not in in those in remission. APN serum concentrations are lower in IBD patients and retinol-binding protein-4 is higher in comparison to healthy controls.
Weigert et al. [144]	2010	310 IBD patients	Chemerin serum levels are elevated in IBD patients in comparison to healthy controls, whereas APN serum levels are higher in UC patients in comparison to healthy controls. CD patients have lower APN serum levels than UC patients, and APN serum level are lower in female CD patients in comparison to female healthy controls.
Biesiada et al. [134]	2012	50 patients with active UC	Serum concentrations of leptin are significantly higher in UC patients with exacerbation of the disease than in patients in remission. The expression of leptin mRNA in colonic mucosa of patients with exacerbation of UC is higher in comparison to those in patients with UC in remission.
Rodrigues et al. [139].	2012	16 patients with ileocecal CD	Serum APN is lower in the active CD patients in comparison to the control, but no differences are seen when comparing the active CD patients to those in remission. APM expression in mWAT is lower in the active CD group in comparison to the control. Serum leptin is similar in all groups.
Chouliaras et al. [138]	2013	50 pediatric IBD patients	In pediatric CD, there is no difference between those in remission and active disease. UC patients in remission have significantly elevated leptin in comparison to those with active disease.
Waluga et al. [145]	2014	40 IBD patients	Serum leptin levels are significantly lower in IBD patients in comparison to healthy controls, and are significantly increased in CD but not UC patients after three months of therapy with corticosteroids and/or azathioprine. Serum resistin and visfatin levels are significantly elevated in IBD patients in comparison to healthy controls. Treatment induces a decrease in the serum resistin concentration only in UC patients and in the serum visfatin concentrations only in CD patients. There are no significant changes in the serum concentrations of adiponectin, chemerin and tissue growth factor-β1 between IBD patients in comparison to healthy controls, and these serum concentrations are not altered by therapy.
Morisaki et al. [157]	2014	63 IBD patients	Serum vaspin concentrations are significantly higher in patients with UC than in patients with CD and healthy controls.
Lu et al. [160]	2014	240 CD patients	Serum omentin-1 levels and colonic omentin-1 expressions are decreased in active CD patients.
Yin et al. [159]	2015	192 IBD patients	Serum omentin-1 levels are significantly lower in both CD and UC patients than in healthy controls.
Terzoudis et al. [149]	2016	120 IBD patients	The chemerin serum is significantly elevated in IBD patients than in healthy controls. Serum visfatin levels in CD patients are significantly higher than in UC patients.
Dogan et al. [153]	2016	31 UC patients	The visfatin serum level is increased in the active UC patients in comparison to post-treatment remission patients and the healthy controls.
Starr et al. [155]	2017	99 pediatric IBD patients	In colonic biopsies from IBD patients, the higher expression of visfatin was observed comparing to controls and there was a correlation between visfatin levels in the colonic biopsies and disease activity.
Kahraman et al. [136]	2017	105 IBD patients	Serum adiponectin levels are significantly lower and leptin is significantly higher in patients with CD and UC.
Ge et al. [148]	2018	24 CD patients	mWAT from CD patients express a higher level of apelin in comparison to controls.
Zuo et al. [163]	2019	24 CD patients	mWAT from CD patients expressed a higher level of Metrnl in comparison to controls.

Crohn’s disease (CD), ulcerative colitis (UC), Adiponectin (APN), mesenteric white adipose tissue (mWAT), meteorin-like (Metrnl).

**Table 3:** Animal studies examining the potential role of adipokines in experimental colitis.

Reference	Year	Study Type	Conclusion
Siegmund et al. [204]	2002	Acute and chronic colitis induced in leptin-deficient ob/ob or WT mice, using DSS or TNBS	In the DSS acute model, ob/ob mice exhibit a 72% reduction of colitis severity and spontaneous release of proinflammatory cytokines from the colon in comparison to WT mice. Replacement of leptin in ob/ob mice converts the disease resistance to susceptibility, indicating that leptin deficiency, not obesity, accounts for the resistance to acute DSS-induced colitis.
Siegmund et al. [206]	2004	Spontaneously developing colitis in leptin-deficient IL-10^−/−^ mice (IL-10^−/−^ ob/ob)	Both IL-10^−/−^ ob/ob and in IL-10^−/−^ mice have a similar degree of intestinal inflammation.
Nishihara et al. [207]	2006	DSS- and TNBS-induced colitis in APN-KO mice	APN-KO mice develop a larger degree of severe colitis in comparison to WT mice. Adenovirus-mediated administration of APN significantly ameliorates the severity of colitis. APN receptors are expressed in intestinal epithelial cells, and APN inhibits LPS-induced IL-8 production in intestinal epithelial cells.
Fayad et al. [211]	2007	DSS- and TNBS-induced colitis in APN-KO mice	APN KO mice are protected from chemically induced colitis; the administration of exogenous APN completely restores the intestinal inflammatory response to DSS.
Han et al. [147]	2007	DSS-induced colitis in C57/BL6 mice and Sprague–Dawley rats	In both mice and rats with experimental colitis, colonic apelin mRNA levels are elevated during DSS-induced colitis.
Teixeira et al. [182]	2011	DSS-induced colitis in C57/BL6 mice	Leptin serum levels are increased in HFD-fed mice in comparison to control and colitis groups. Leptin expression in adipose tissue is elevated in both HFD groups in comparison to the colitis (normal-diet) group.
Arsenescu et al. [210]	2011	DSS-induced colitis in C57/BL6 mice	Adenovirus-mediated administration of APN ameliorates the severity of DSS-induced colitis. The APP homolog osmotin similarly reduces colitis severity.
Saxena et al. [208]	2012	DSS-induced colitis in APN-KO mice	APN deficiency exacerbates the severity of DSS-induced colitis and increases the production of proinflammatory cytokines. In WT mice in DSS-induced colitis. There is a decrease in the serum adiponectin level in comparison to the control.
Singh et al. [133]	2013	Spontaneously developing chronic colitis in IL-10^−/−^ mice	Pegylated leptin antagonist ameliorates the development of chronic experimental colitis.
Sideri et al. [128]	2015	TNBS-induced colitis in C57/BL6 mice	Silencing adiponectin receptor 1 exacerbates TNBS-induced colitis in mice.
Kaur et al. [212]	2015	DSS-induced colitis in C57/BL6 mice	APN KO mice are less susceptible to DSS-induced colitis than WT mice and have a reduced release of proinflammatory cytokines.
Bilski et al. [185]	2015	TNBS-induced colitis Sprague–Dawley rats	The impaired healing of colitis observed in rats fed the HFD is accompanied by an increase in in leptin but also the reduction in adiponectin plasma levels.
Mazur-Bialy et al. [184]	2017	TNBS-induced colitis in C57/BL6 mice	There is increased leptin and decreased adiponectin plasma levels and elevated leptin and decreased adiponectin expression in adipose tissue, which correspond to disease exacerbation in HFD animals.
Obeid et al. [209]	2017	DSS-induced colitis in APN-KO mice	APN-KO mice which have shown an aggravation of DSS-induced colitis have a greater inflammatory cell infiltration and higher presence of activated B cells in comparison to controls, accompanied by an elevated proinflammatory cytokine profile production.
Ge et al. [148]	2018	Spontaneously developing chronic colitis in IL-10^−/−^ mice	Apelin significantly ameliorates chronic colitis in Il-10^−/−^ mice, demonstrated by the decreased disease activity index, inflammatory scores, and decreased levels of proinflammatory cytokines.
Zuo et al. [163]	2019	Spontaneously developing chronic colitis in IL-10^−/−^ mice	In IL-10^−/−^ mice with spontaneous colitis, administration of metrnl decreases pathological alterations in mWAT, increases adipocyte size, and ameliorates inflammation.

Adiponectin (APN), adiponectin-knockout (APN-KO), dextran sulphate sodium (DSS), wild-type (WT), lipopolysaccharide (LPS), trinitrobenzene sulfonic acid (TNBS).
